# A Rare Case of Pseudomembrane-Associated Ulcerative Colitis

**DOI:** 10.7759/cureus.33152

**Published:** 2022-12-30

**Authors:** Mohammad A Ahmed-Khan, Kayvon Moin, Carly M Funk, Felipe Carmona-Pires

**Affiliations:** 1 Internal Medicine, Danbury Hospital - Yale School of Medicine, Danbury, USA; 2 School of Medicine, American University of the Caribbean School of Medicine, Cupecoy, SXM

**Keywords:** infectious disease, general gastroenterology, clostridioides difficile infection, pseudomembrane, ulcerative colitis

## Abstract

Ulcerative colitis (UC) is a chronic, life-long inflammatory bowel disease that normally presents with bloody diarrhea, fever, abdominal pain, and leukocytosis. Diagnosis is usually based on clinical presentation, endoscopy with biopsy, and exclusion of alternative diagnoses. In very rare cases, pseudomembranes may be found on colonoscopy in patients with an early UC flare. Historically, the objective finding of pseudomembranes has been exclusively used to diagnose a *Clostridioides difficile* infection (CDI); however, diagnostic testing must be correctly utilized to confirm whether a CDI is truly the cause of the presence of pseudomembranes, and not an alternative etiology, such as UC.

In this case, we discuss a 43-year-old female who presented to the hospital with worsening chronic bloody diarrhea after being seen in the outpatient clinic for a questionable CDI. She underwent endoscopic evaluation revealing pseudomembranous colitis; however, *C. difficile* testing showed one positive gastrointestinal (GI) pathogen panel and multiple negative antigens and toxin enzyme immunoassays (EIA). With a clinical suspicion of early UC, the patient was treated with mesalamine enemas and improved clinically before discharge. Several months later, she underwent endoscopic evaluation with biopsy, which showed findings consistent with a diagnosis of UC.

## Introduction

UC is a debilitating, chronic, immune-mediated process that is on the spectrum of inflammatory bowel diseases affecting the large intestine. It is associated with a relapsing and remitting course. Initially, it affects the rectum and then spreads proximally to the colon and other parts of the large intestine [1-4]. The overall prevalence within the United States has climbed in recent years, affecting over 600,000 people [2]. Presentation occurs at any time; however, there is a bimodal distribution typically between ages 15 and 30 and after 50 [1,3]. Diagnosis is based on symptoms, endoscopy with biopsy, and the absence of alternative diagnoses such as infectious bacterial, amebic, or viral, Crohn's disease, ischemic colitis, etc. Patients initially present with left lower quadrant pain, bloody diarrhea, urgency, and tenesmus [1,5]. On gross examination, the mucosa is friable and granular in appearance with superficial ulceration [6]. In a biopsy specimen, extensive crypt distortion is seen along with transmucosal inflammatory infiltration causing crypt abscesses [6]. 

Similarly, another inflammatory condition of the colon is pseudomembranous colitis, which causes yellow-white plaques on the mucosa that coalesce into pseudomembranes [7]. The disease is most commonly a result of a severe CDI [8]. Inflammation, disruption of cytoskeletons, injury to signaling pathways, and eventual colonic cell death are caused by *Clostridioides difficile* exotoxins A and B [9]. In turn, tight junctions between neighboring colonic cells are disrupted, allowing neutrophils to invade the mucosa and activate the body's native immune system. Epithelial surface neutrophils form pseudomembranes as a result [10]. However, pseudomembranous colitis is not exclusively caused by *C. difficile*, it has been linked to other microbes, parasites, viruses, and chemicals [11]. It has more recently been seen in the prodromal phase of patients with UC [12,13]. Our case describes a 43-year-old patient who presented to the emergency department with fever, vomiting, and bloody diarrhea, and who was found to have pseudomembranes on endoscopic evaluation in the setting of three negative *C. difficile* antigen/toxin enzyme immunoassays (EIA).

## Case presentation

A 43-year-old female with a relevant past medical history of gastroesophageal reflux disease (GERD) and peptic ulcer disease (PUD) was seen at the outpatient gastroenterology office due to bloody diarrhea several times per day for a duration of four weeks. She also reported left-sided abdominal cramping and fatigue. She denied any pertinent family history, recent antibiotic use, new medications, fever, chills, nausea and/or vomiting, or recent travel. Her only medications at the time were pantoprazole for chronic GERD and desogestrel-ethinyl estradiol for menopause. A stool culture of the diarrhea was obtained and was analyzed via GI polymerase chain reaction (PCR) panel and *C. difficile* EIA. The GI panel was positive for *C. difficile* and negative for all other infectious etiologies; the EIA was negative for *C. difficile* antigen (glutamate dehydrogenase) toxin. *C. difficile* colitis was diagnosed and the patient was prescribed vancomycin 125 milligrams four times daily for 14 days. The patient had a similar presentation two years prior, in which a colonoscopy was ordered. The patient underwent the colonoscopy seven months after the order was placed, at a time in which her symptoms had self-resolved. The findings at the time were significant for a hyperplastic polyp and a pseudopolyp in the cecum and recto-sigmoid colon, respectively. The patient was recommended a follow-up colonoscopy in five years.

Three days after starting vancomycin, the patient came into the emergency department (ED) with complaints of worsening symptoms. The patient had also developed fever, chills, nausea, vomiting, severe left-sided abdominal pain, and bloody diarrhea with a frequency of 10-12 bowel movements per day. Abdominal exam findings showed a flat abdomen with left-sided periumbilical tenderness without guarding and normal bowel sounds. No masses or organomegaly were palpated. Initial laboratory findings were significant for a leukocyte count of 23.4 × 10⁹/L with a neutrophilic predominance and potassium level of 3.4 mmol/L. A CT scan obtained to rule out diverticulitis showed diffuse colonic thickening, consistent with infectious or inflammatory pancolitis. A repeat *C. difficile* EIA was negative once again. Vancomycin, metronidazole, and pain management with hydromorphone were ordered for the patient, and the patient was admitted to the hospital, pending a colonoscopy. Additional lab findings showed an elevated C-reactive protein (CRP) level of 129.8 mg/L, erythrocyte sedimentation rate (ESR) of 23 mm/hour, and calprotectin level of 1900, making inflammatory bowel disease (IBD) a likely etiology to the patient’s presentation. 

**Figure 1:**
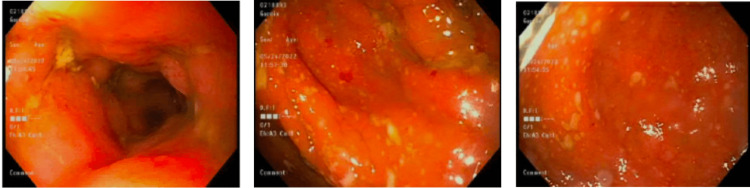
Colonoscopy findings showing pseudomembranous exudate in the transverse colon (left), descending colon (middle), and sigmoid colon (right).

Surprisingly, the colonoscopy report showed a diffuse mucopurulent pseudomembranous exudate from the rectum to the transverse colon with moderate active colitis, confirmed by biopsy (Figure 1). Stool was also collected during the procedure for analysis, which again showed a negative *C. difficile* antigen/toxin via EIA. The pathologist reported that the inflammation seen on the biopsies was very non-specific, but could possibly be the initial changes seen in IBD. The patient was started on mesalamine enemas and was kept on the antibiotic regimen. Over the next few days, her bowel movement frequency decreased to three to four times per day, and laboratory findings ultimately normalized. The patient was discharged on a two-week course of oral metronidazole and vancomycin as well as oral mesalamine and was instructed to return to outpatient GI for a follow-up repeat colonoscopy in eight weeks.

The patient returned for her repeat colonoscopy a few months later. In the time between discharge and this procedure, the patient had a period of complete resolution of symptoms and stopped all medications. Shortly after, she started to develop similar symptoms as she did when she was hospitalized and was instructed to schedule the colonoscopy as soon as possible. Interestingly, the colonoscopy showed a diffuse area of moderately congested, erythematous, inflamed, and ulcerated mucosa in the entire colon. Biopsies confirmed the diagnosis of UC, and the patient was prescribed oral mesalamine two 1.25g tablets per day, and was instructed to return for routine follow-up and management. 

## Discussion

Although the official diagnosis of ulcerative colitis was not established until the most recent outpatient colonoscopy procedure, there was high clinical suspicion of this diagnosis from her initial colonoscopy two years before the ED visit. At the time of the first colonoscopy, the patient’s episode of diarrhea with hematochezia had subsided, and a pseudopolyp was found in the recto-sigmoid colon. Although pseudopolyps are only found in about 30% of UC patients [14], the presence of one on colonoscopy should clue in the possibility of this diagnosis. The pseudomembranes found at the time of the second colonoscopy during the patient’s admission, unfortunately, guided the patient’s treatment plan towards *C. difficile*-induced pseudomembranous colitis due to the incorrect assumption that* C. difficile* and pseudomembranous colitis are strictly interchangeable diagnoses. As a result of elevated calprotectin, ESR, and CRP levels and the clinical presentation, mesalamine enemas were administered for suspected UC showing clinically appreciable improvement.

It is most likely that the patient had UC-associated pseudomembranous colitis because three separate EIAs for *C. difficile* antigen/toxin were negative. Although the initial GI PCR panel of the patient's diarrheal stools was positive for *C. difficile* and was the reason for continuous treatment for suspected *C. difficile*, the statistical significance of these diagnostic tests should be noted. The GI PCR panel is included in the nucleic acid amplification test (NAAT), which has been used primarily as a screening tool for toxigenic strains due to its sensitivity of 97.1% [15]. Toxigenic strains can be detected with this diagnostic test but it fails to detect toxins. Thus, these characteristics fail to differentiate between CDI and asymptomatic carriers, causing an overdiagnosis of CDI [16,17]. EIA for the *C. difficile* antigen and toxins A and B are usually used for confirmatory testing after a positive GI PCR panel due to its high specificity of 99% [18,19]. As a result, we can assume the GI PCR panel was a false positive or that the patient was an asymptomatic carrier based on the three confirmatory negative EIAs.

Current literature on the association between pseudomembranes and UC in the setting of an absence of CDI is fairly limited. One case report describes a patient who was positive for *C. difficile* toxin and was found to have pseudomembranes on colonoscopy, but after antibiotic treatment, was found to have underlying IBD-associated inflammation with a resolution of the pseudomembranes on repeat colonoscopy [12]; However, our patient was found to have pseudomembranes on colonoscopy with a deteriorating clinical presentation despite being on antibiotics for nearly a week. Our patient only started to improve symptomatically when mesalamine enemas were added to her management. It is unlikely that our patient was suffering from a concurrent CDI and a UC flare.

## Conclusions

This case report has shed important light on the misconception that pseudomembranous colitis is only associated with CDI. Our case as well as past literature, including case reports supported by endoscopic imaging and histopathologic evaluations, demonstrates that UC flare can also be the culprit. Appropriate diagnostic testing and focused endoscopic evaluation must be performed on patients with symptoms as described in our case to ensure there is no delay in the appropriate management of UC. Our hope with this case report is to bring awareness to medical providers about the possibility of UC or an alternate diagnosis when encountering pseudomembranes in the absence of a definitive CDI.
